# “University students’ economic situation during the COVID-19 pandemic: A cross-sectional study in Germany”

**DOI:** 10.1371/journal.pone.0275055

**Published:** 2022-10-06

**Authors:** Sandra Claudia Gewalt, Sarah Berger, Regina Krisam, Johannes Krisam, Markus Breuer

**Affiliations:** 1 SRH University Heidelberg, Heidelberg, Germany; 2 Centre for Postgraduate Nursing Studies, University of Otago, Christchurch, New Zealand; 3 Institute of Medical Biometry, University Hospital Heidelberg, Heidelberg, Germany; King Abdulaziz University, SAUDI ARABIA

## Abstract

The COVID-19 pandemic caused a major economic downturn that disproportionally affected university students. This empirical research investigated effects and risk factors of the pandemic on students’ economic situation with focus on financial distress and financial limitations. Data was collected using an online survey in May and June 2020 from students (n = 917) enrolled at universities in Germany. 80.6% were enrolled in bachelor programs (n = 738), the mean semester was 3.8 (standard deviation (SD = 2.0) and students’ mean age was 23.1 years (SD = 4.1). 51.8% (n = 472) were female and 47.4% (n = 432) male. 56.7% (n = 506) of students worked before the pandemic. More than one third reported a decrease in income (36.5%; n = 334) and an increase in financial constraints (38.7%; n = 354). A multivariate logistic regression analysis showed that students with regular income were less likely to experience financial distress compared to those without (odds ratio (OR) = 0.456; p = 0.014). Furthermore, working part-time as associated with a higher financial distress compared to those without part-time employment (OR = 1.811; p = 0.003). Students who worked part-time before the pandemic also had a higher probability of increased financial restriction (or constraint) compared to those who did not work part-time (OR = 2.094; p < 0.001). University students were disproportionally affected by the economic consequences of the COVID-19 pandemic, which increased students’ economic uncertainty. To offset such problems, financial aid schemes for students need to be made available to alleviate distress and to allow students to focus on their studies but should not compound problems by leading to financial hardship at a later point in time.

## Background

The COVID-19 pandemic caused a major economic downturn. Vulnerable groups, including university students, were especially affected by the black swan event. Public health measures, including temporary lockdowns, caused a standstill of non-essential activities. A multitude of business processes were halted and contact restrictions, strictly limiting the number of people per gathering, were introduced. Closures of educational institutions internationally impacted nearly 70% of the world’s student population [1]. Imposed measures to limit COVID-19 transmission restricted the freedom of individuals in unprecedented ways. Public health measures restricting individual freedoms and economic uncertainty are two key factors linked to adverse economic situations for university students.

### Students’ economic challenges during the current pandemic

The pandemic driven economic downturn created economic challenges for university students due to a decrease or even loss of income. A recent review found that social distancing, self-isolation and travel restrictions led to a loss of various jobs, especially in the service industry [2]. Some college and university students lost their part-time jobs when regional businesses were closed [3]. A study of the National Bureau of Economic Research with more than 10,000 respondents highlighted that roughly 50% of respondents stated income and wealth losses [4]. Total consumer spending decreased 31 log percentage points, with travel and clothing seeing the largest decreases. The introduction of lockdowns may have caused much of the decline in employment in subsequent months, as well as a decline in consumer spending [4]. A population-based sample of students in New York city found that most students reported a loss of household income and half reported worries about losing housing [5]. Household spending was reported to have declined by 40 to 50% during the COVID-19 pandemic and income reductions have become much more common, with a mean decline of around 30% [6]. Low-wage workers experienced much larger job losses that lasted for several months compared to high-wage workers [7]. According to the Germany National Association for Student Affairs, the majority of students in Germany were employed prior to the onset of the pandemic to cover living costs and university fees: 64% of all students represented in the study (i.e. before the pandemic) were employed, whereas only 5% were de facto full-time employees and 34% were working one to two days per week [8]. An earlier study found that 66% of undergraduate students worked during their studies; for half of them the part-time job was necessary to support themselves financially [9]. Most performed simple temporary jobs such as working as a waiter, taxi driver, sales assistant, or clerical assistant, 28% worked as a student research assistant and 11% as a tutor [8]. Precarious employment has been identified as risk factor for poor mental health of young individuals [10]. A recently published study on Spanish workers identified the need for interventional and preventive programs targeting mental health in economic crisis scenarios such as the current pandemic and highlighted the relevance of introducing social and income policies to prevent mental health problems [11]. Equally, recent findings from Argentina argue that countries’ policies should be supportive to workers facing economic hardships and unemployment to counterbalance the negative impact on mental health caused by the current pandemic [12]. With many temporary jobs (for example in the service industry) being lost, students were likely to have experienced negative financial effects. During the pandemic, evidence suggests an elevated number of students may have experienced economic hardship due to decreased or loss of income.

Despite such negative financial impacts on students during the pandemic, they still had to meet their living costs and fees associated with their studies. According to the Federal Ministry of Education and Research in Germany, the cost of food, accommodation, clothing and cultural activities were above the European Union average [13]. Predominant cost drivers included living costs, university tuition fees, semester fees and health insurance. Monthly living costs typically included rent, food, clothing, books, telephone, semester and tuition fees and were identified to add up to 867 Euro on average [13]. The German consumer price index rose by 0.5% on annual average in 2020 compared with 2019 [14]. Yet, in November 2021, compared to the same month a year earlier, it rose by 5,2% [15]. Semester and tuition fees still had to be covered by university students. These varied considerably. In the Rhine-Neckar region in Germany, public universities (of applied sciences) charged approximately 350–380 Euro per semester. Private institutions charged annual university fees of approximately 7,800 Euro for a bachelor’s degree and around 9,600 Euro for a master’s degree. Universities (of applied sciences) offering co-delivered apprenticeship education programs charged roughly 365 Euro per annum, whilst the employer provided an annual income of about 18,000 Euro. On-going living costs and tuition expenses during the COVID-19 pandemic compounded financial challenges in the vulnerable group of university students many of whom were dealing with income decreases or loss at the same time.

### Students’ distress during the current pandemic

Whilst the COVID-19 pandemic posed an immediate risk to university students’ short and long-term physical health [16], there is also evidence that it aggravated students’ stress levels. Negative stress can occur when environmental demands overwhelm or surpass individuals’ coping capacities [17]. Poor mental health can be linked to rapid social change, stressful work requirements, social exclusion, unhealthy routines and physical illness [18]. Findings of an analysis of representative data of active members of the workforce of six European nations indicated a significant relationship between immediate economic hardships during the COVID-19 lockdown and feelings of depression and anxiety [19]. Furthermore, underprivileged college students were more likely to show mental health symptoms such as anxiety, low self-esteem and depression [20]. Recent studies revealed increased stress levels and anxiety in Indian medical students due to excessive responsibilities and insufficient resources to counterbalance during the current pandemic [21]. A cross-country comparative study with Indonesian, Taiwanese and Thai university students revealed the need for special attention to Thai university students due to observed high levels of anxiety as well as the demand for a good support system for university students [22]. Economic burden, identified via self-reported financial problems, was associated with adverse mental health outcomes in Thailand [23]. Continuous socio-economic pressures, such as those caused by the pandemic, potentially posed a risk to students’ mental health and well-being. Research on university students in Germany found that pandemic-related social restrictions caused a decrease in wellbeing in the majority of students [24]. A population-based study identified that financial hardship, unemployment and major life events were strongly and independently related to mental health symptoms [25]. High levels of food and housing insecurity were the strongest predictors of students’ anxiety and depression [5]. A rapid review on the effects of the pandemic on mental health identified mostly negative psychological effects, including post-traumatic stress symptoms and the fear of financial loss [26]. A study on the needs of students in the United States of America showed that students faced increasing housing and food insecurity, economic hardship, a lack of social-cultural connectedness and sense of belonging as well as uncertainty about the future [27]. Furthermore, students lost jobs, internships or job offers and some forecasts indicated this would also impact earning potential later in life (at age 35) [28]. Students with lower income were 55% more likely than their higher-income peers to have to delay graduation due to COVID-19 [28]. Students in their final year were concerned about the job market that they would be entering [3]. The COVID-19 pandemic created multiple challenges for students including both health-related and economic stressors.

### Gender-related differences

During the COVID-19 pandemic, women generally reported more negative emotional experiences than their male counterparts [29]. Although it was observed that men were more likely to experience negative health-related consequences from COVID-19, women reported higher levels of anxiety and more negative expectations compared to men [29]. A recent study on COVID-19 and its effects on university students’ mental health in Switzerland found that female students showed to have poorer mental health after controlling for different levels of social inclusion and pandemic-related stressors [30]. These results were not surprising, as research on gender differences in relation to stress showed that women experienced more stress than men [31]. A study of stress among medical students related to an outbreak of Middle East Respiratory Syndrome (MERS) identified that female students had a significantly higher mean stress level than male students [32]. Moreover, a recent longitudinal study of the mental health of Chinese adults found that female sex was associated with higher psychological effects [33]. A study on suicide rates during the current pandemic showed an increase during the second wave in 2020, with a larger increase among females [34]. Researchers investigating gender, debt and college drop-out rates found that both women and men experienced a slower, or even decreasing, likelihood of graduation when bearing large amounts of debt [35]. Yet, recent research from North America found that women were more optimistic about the financial consequences of the pandemic than men [29]. Research findings indicate gender-related differences in experiences of mental health stress and financial difficulties and this is consistent in the context of the COVID-19 pandemic.

Through a literature search, it was determined that there were no published studies on the effects of the COVID-19 pandemic on the financial situation and distress of students in the Rhine-Neckar region in Germany. Similarly, no studies were available from on students’ gender-specific differences in the Rhine-Neckar region in Germany. Therefore, this study examined how the COVID-19 pandemic affected university students’ economic situation with focus on financial distress and financial constraints and sought to analyse changes in the students’ economic situation, with respect to income and consumption.

## Methods

This empirical study was based on a cross-sectional study design using an online survey tool (taking less than five minutes to complete). Data was collected anonymously via a neutral and independent service provider. Each IP (internet protocol) address, could only access the online survey once. This measure prevented double entry from the same IP address. Duplicate entries were avoided by preventing participants with the same IP address to access the survey twice. No cookies were used to assign a unique user identifier to a participant’s computer. Receiving only the responses by the external service provider, there is no information on the view rate (ratio of unique survey visitors/unique site visitors) or on the participation rate (ratio of unique visitors who agreed to participate/unique first survey page visitors). There was no log file analysis for identification of multiple entries. Data collection was undertaken over five weeks from the end of May to the end of June 2020 in the Rhine-Neckar region in Germany. The lockdown in Germany reached its peak in March and April 2020. However, everyday life remained significantly affected by restrictions in May and June 2020.

### Recruitment

The study population in the Rhine-Neckar region was selected using convenience sampling. The Rhine-Neckar region in Germany is special as it includes a high density of student population. It combines the cities Heidelberg, Mannheim and Ludwigshafen. All cities dispose of public and private universities (of applied sciences). The respective registry offices of the eight universities (of applied sciences) were contacted and asked to send an invitation email to all currently enrolled students. The email contained a brief description of the purpose of the survey and an electronic link. A flyer was created and attached to this email. In addition, an invitation with an overview of the survey and a link to the survey was published on social media channels of the universities (of applied sciences) and the student councils for each university. In a second wave of recruiting, a reminder was sent via social media channels that were accessed by students in the Rhine-Neckar region. Follow-up data was not collected. There was no number of questionnaire items per page or number of pages as participants could scroll down to access further questions. A moving bar on the side of the online survey indicated the progression within the survey. The survey was designed as open survey which allowed participation without password protection. There was no initial contact with study participants via the internet prior to a participation. Participants were not able to review or change their answers (eg. via a back button). We did not apply randomization of items or adaptive questioning. There was no timeframe that was used as a cut-off point.

### Participants

Students enrolled at one of the eight universities as well as universities of applied sciences in the three cities of the Rhine-Neckar region, including Heidelberg, Mannheim and Ludwigshafen, were recruited. The overarching goal was to involve the entire student body at the eight universities and universities of applied sciences in the Rhine-Neckar region. Inclusion criteria were enrolment at one of the eight universities or universities of applied sciences in the Rhine-Neckar region and basic knowledge of English. The invitation to fill out the survey and the survey itself were only given in English. Participation in the study was voluntary and could be ended at any time without risk of negative consequences. As an incentive, five gift vouchers with a value of 10 euros per voucher were randomly drawn and given to participants.

### Variables

The primary aim of this survey was to investigate short-term COVID-19-related effects on students, resulting in two primary outcomes, **financial distress** and **financial limitations**

Due to the challenges in measuring mental health, financial distress stress levels reported by university students were measured from zero to ten on the basis of the question "Select your level of negative stress (…) 0 = no stress, 5 = medium stress, 10 = highest level of stress”. To compare students who reported having no to low financial distress (from 0 to 4) with students who reported having moderate to high financial distress (from 5 to 10), a binary variable was derived.

Increased financial limitations were assessed by asking the question “How much have your financial limitations increased since the COVID-19 pandemic?”. This outcome was captured on a five-point Likert scale ranging from “not at all”, “very little”, “a little”, “much”, and “very much”. Again, to compare students who reported having no increased financial limitations (“not at all”) with students who reported at least “very little” increased financial limitations, a binary variable was derived. In order to describe the economic situation during the COVID-19 pandemic, participants were asked for information with regard to

- income changes,
- stress due to difficulties in paying rent,
- decrease in weekly spending,
- plans to decrease spending in the next weeks or months,
- change in spending money on healthcare,
- postponing purchases to the future.

Potential confounder variables were also collected. These were gender, age, university location, field of study (business, health, information technology, MINT (mathematics, computer science, science and technology / technical) and others), degree, semester, education, grade point average, income and employment before the pandemic were collected.

### Data sources/measurement

The self-designed questionnaire was established based on a comprehensive literature review and was designed striving for a maximum of content validity. Data was collected on students’ financial situation as negative stress. Students could select among closed-ended questions related to the location of their university (of science), degree, study subject, semester, age, gender, financial parameters including income and purchasing behaviour before and after the onset of the pandemic as well as levels of distress and postponement of purchases.

### Bias

Selection bias was minimized by contacting registry offices of the eight universities (of applied sciences) and asking them to send an invitation email to all currently enrolled students. Yet, due to conducting the survey in English language only, a selection bias might have occurred. This might have caused a higher participation in international students and those who perceive to have a good command of English.

### Sample size

We expected that about 900 participants would return a fully answered online questionnaire. With this sample size at hand, we expected that we could develop a logistic regression model that could provide us with adequately precise 95% confidence intervals for the odds ratios of predictor variables of financial distress and financial limitations, as well as an adequately precise 95% confidence interval for the area under the curve (AUC) to measure the overall predictive strength of our model. Assuming that between 25%-75% of all respondents were having at least a moderate level of financial distress/at least very little increased financial limitations, the maximal width of the 95% confidence interval of the AUC with a sample size of 900 respondents amounts to 8.7 percent points (assuming the lowest possible AUC of 0.5), representing an adequately precise estimation of the AUC of the logistic regression models. The sample size calculation was done using the software PASS v16.0.12.

### Statistical analysis

All continuous variables were summarized by number of non-missing values, mean, standard deviation, median, first quartile (25^th^ percentile), third quartile (75^th^ percentile), minimum and maximum. For binary and categorical variables, absolute and relative frequencies were provided. The number of missing responses, if any, was reported as a separate category. We checked responses for completeness after the questionnaires had been submitted. Percentages were based on all non-missing values (= 100%). In order to examine differences between students with moderate to high stress compared to students with no or low stress, univariable statistical tests were conducted with regard to differences in the distribution of risk factors. Using t-tests for continuous variables, Mann-Whitney u-tests for ordered categorical variables or chi-square tests for categorical variables. Analogously, univariable statistical tests were used to identify differences in risk factor distributions for students with no increased financial limitations compared to students with at least “very little” financial limitations. Furthermore, the association between the primary and secondary economic outcomes was investigated by means of univariable statistical tests. Also, gender-based differences with regard to economic outcomes were evaluated using univariable statistical tests. Besides the univariable analyses, multivariable logistic regression models were fitted to estimate the influence of risk factors on the two primary economic outcomes. All relevant variables were included in the model and no variable selection was done by the model. No weighting of items or propensity score methods were used. Odds ratios were calculated together with 95% confidence intervals and p-values. The AUC was calculated with a 95% confidence interval as well to estimate the predictive quality of the model. Possible multicollinearity was checked by determining variance inflation factors (VIFs), for which we assumed that a VIF larger than 5 was problematic [36]. No subgroup analyses were done. Missing values were not imputed. No sensitivity analyses were done. Statistical analysis was done via R (version 4.2.1, http://r-project.org), where the pROC package [37] was used for determining AUCs, and the car package [38] was used to determine VIFs. P-values ≤0.05 were regarded as statistically significant. Due to the exploratory nature of the study, derived p-values could only be interpreted descriptively.

The completed the STROBE (Strengthening the Reporting of Observational Studies in Epidemiology) Checklist and CHERRIES (Checklist for Reporting Results of Internet E-Surveys) is attached as S1 Checklist.

### Ethics approval

Ethical approval for the research project "Impact of the COVID-19 pandemic on students of the Rhine-Neckar district with a focus on health, finances and learning. A cross-sectional study was obtained by the Joint Ethics Committee of the Heidelberg University of Education and the SRH University Heidelberg.

## Results

### Participants

1.245 participants started and 917 participants completed the survey. Thus, the completion rate was 73.65%. The collective of 917 students who completed the survey in full, was used for statistical analysis. Among those 917, questions which were not answered, were marked as missing. The total of participants who were asked to fill the self-administered online survey is not known as the invitation to participate was sent to registry offices of the eight universities (of applied sciences) who sent invitation emails to all currently enrolled students. Due to data protection of students’ identity, this was the procedure of choice. Information on reasons for study drop-out or non-participation was not collected.

### Baseline demographics

27.0% (n = 247) of students were enrolled in Heidelberg, 25.1% (n = 229) in Ludwigshafen and 47.9% (n = 438) in Mannheim (914 of 917 respondents). Most students (80.6%; n = 738) were enrolled in a bachelor’s degree. 15.9% (n = 146) were enrolled in a master’s degree, 1.0% (n = 9) pursued a state examination and 2.5% (n = 23) were doctoral students (916 of 917 respondents). As there were only few doctoral students and students pursuing state examination, these were excluded from the univariable and multivariable analyses, which focussed only on Bachelor and Master students. 42.8% (n = 392) of students were pursuing business studies, with MINT / technical 16.7% (n = 153), information technology 16.3% (n = 149) and health 16.2% (n = 148). The “others” category comprised 8.1% (n = 74) of students (916 out of 917 respondents). Mean semester was 3.8 (SD = 2.03; 896 out of 917 respondents). Students’ mean age was 23.1 years (SD = 4.08; 902 out of 917 respondents). 51.8% (n = 472) identified as female and 47.4% (n = 432) identified as male. Only 0.9% (n = 8) students identified as diverse (912 out of 917 respondents), which lead us to exclude these students from the univariable and multivariable analyses, focusing only on students who identified themselves as male or female.

### Students’ economic situation prior to the pandemic

Prior to the onset of the pandemic in Germany, 38.8% (n = 324) had a regular income from a (student) job. 12.0% (n = 100) received regular financial support from their family. 2.8% (n = 23) received income from the student loan scheme “BAföG” *Bundesausbildungsförderungsgesetz*, i.e. Federal Education and Training Assistance Act, Germany’s Federal Study Assistance, 8.4% (n = 70) had regular income from a loan (from a commercial bank). 8.4% (n = 70) declared they had no income and lived off savings (834 out of 917 respondents). Details can be found in Table 1 of the S1 File. For the univariable and multivariable analyses, the income was simplified to a variable with three categories (no income/ irregular income/ regular income).

Most students, namely 56.7% (n = 506), held a part-time job prior to the pandemic (see Table 2 in S1 File). Almost one in five students worked more than 20 hours per week (18.8%; n = 168; 893 out of 917 respondents). This can be explained by students who were enrolled in dual study apprenticeship programs, allowing them to alternate study and work in their trade or occupation.

### Outcome data

**Fig 1:**
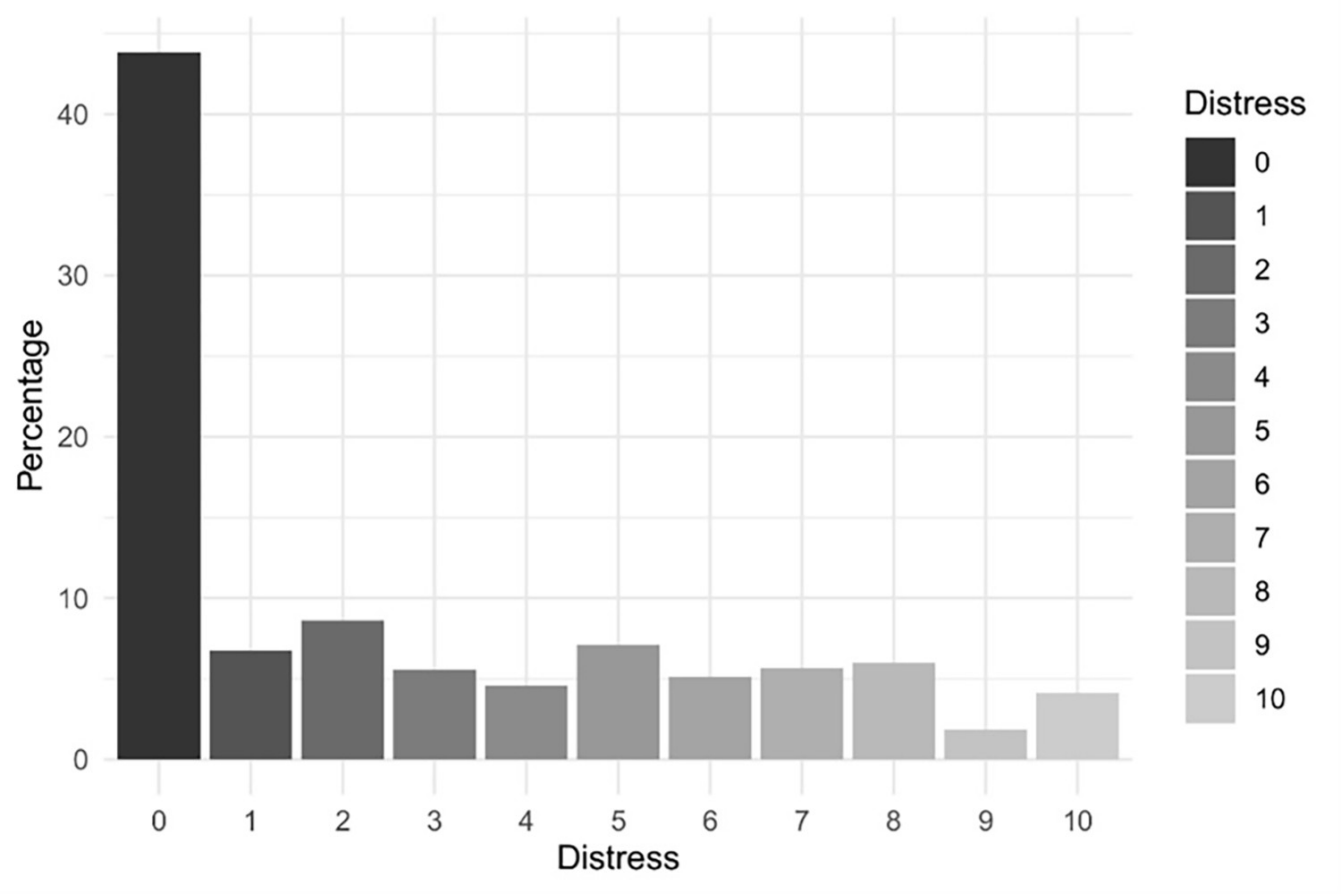
Distress due to financial difficulties.

Mean levels of distress related to financial difficulties were 2.7 (SD = 3.20). A bar plot illustrating the self-perceived stress distribution is shown in Fig 1.

A total of 274 (30.1%) of participants reported having moderate to high stress (from 5 to 10). Participants reported changes in their economic situation due to the COVID-19 pandemic. 287 (31.3%) respondents reported at least a little increase in financial limitations. More than one third (36.5%; n = 334) declared their income had decreased due to the pandemic. Of these, 9.7% (n = 89) reported their income had fallen very sharply. More than one third, 38.6% (n = 354) said their financial constraints had soared and 7.2% (n = 66) reported they had soared significantly (916 out of 917 respondents). Details are found in Table 1.

**Table 1:** Change in financial limitations, income, and weekly spending.

	How much have your financial limitations increased since the COVID-19 pandemic? n (%)	How much did your income decrease during the COVID-19 pandemic? n (%)	How much have you decreased your weekly spending due to the current pandemic? n (%)
very much	66 (7.2%)	89 (9.7%)	122 (13.3%)
much	71 (7.8%)	74 (8.1%)	249 (27.2%)
a little	150 (16.4%)	112 (12.2%)	300 (32.8%)
very little	67 (7.3%)	59 (6.4%)	82 (9.0%)
not at all	562 (61.4%)	581 (63.5%)	163 (17.8%)
missing	1	2	1

In terms of students’ consumption, 82.3% (n = 753) reduced their weekly spending (see Table 1). 13.3% (n = 122) declared that they had significantly reduced their weekly expenditures (916 out of 917 respondents).

Changes in students’ consumption were mainly due to lower spending in the categories “holidays”, “restaurants”, “transportation” and “fashion” (see Table 2): With 87.0% (n = 793) the vast majority spent less on holidays (911 of 917 respondents). 82.6% (n = 752) spent less on restaurants (910 of 917 respondents). 66.5% (n = 606) spent less on transportation by car or train (911 of 917 respondents). More than half of the students (55.5%, n = 507) reported spending less on fashion, including clothing and shoes, since the start of the pandemic.

**Table 2:** Change in spending.

Do you spend more or less money on/in … since the COVID-19 pandemic? n (%)	more	same	less	missing
Holidays	7 (0.8%)	111 (12.2%)	793 (87.0%)	6
Restaurants	48 (5.3%)	110 (12.1%)	752 (82.6%)	7
Transportation by car/ train	68 (7.5%)	237 (26.0%)	606 (66.5%)	6
Fashion (clothing and shoes)	98 (10.7%)	308 (33.7%)	507 (55.5%)	4
Healthcare (medicine, consultation, treatment, etc.)	74 (8.1%)	673 (73.6%)	168 (18.4%)	2
Supermarkets	316 (34.5%)	459 (50.2%)	140 (15.3%)	2
Subscription of online media	133 (14.6%)	695 (76.1%)	85 (9.3%)	4

A rather stable consumption pattern was found in the categories “healthcare”, “supermarkets” and the “subscription of online media” (including newspaper and entertainment services): Students’ healthcare consumption (including medicine, consultation and treatment) remained rather unchanged: 73.6% (n = 673) spent the same amounts (915 of 917 respondents). While every second student (50.2%; n = 459) had spent the same amount in supermarkets since the start of pandemic, a third (34.5%; n = 316) increased spending in supermarkets (915 of 917 respondents). Expenditure on subscribing to online media (including newspaper and entertainment services) remained the same for most students (76.1%; n = 695).

In terms of future purchases, every second student (50.3%; n = 459) reported postponing purchases to a future date due to the pandemic. One in five students (21.8%; n = 199) reported postponing any purchase that was not strictly necessary, such as food and grocery shopping (913 out of 917 respondents). Details can be found in Table 3 in the S1 File.

More than half of the respondents (54.8%; n = 495) wanted to spend less in the next few weeks or months (902 of 917 respondents, see Table 4 in the S1 File).

Almost every second student (45.3%; n = 412) planned to give up holidays in 2020. 29.0% (n = 264) indicated this was due to health-related risks of the pandemic and 16.3% (n = 148) to save money (910 of 917 respondents).

Perceptions of the future showed a predominance of negative expectations. The onset of the COVID-19 pandemic was a period of significant change and uncertainty for students. More than half of the study participants, 54.8% (n = 500), believed that the pandemic would have negative effects on internships, job search and employment opportunities in the future. Among these, 15.6% (n = 142) expected severe negative consequences and 39.2% (n = 358) expected negative consequences (913 of 917 respondents).

### Risk factors for students’ distress

Risk factors for students with no or little negative stress were compared to students with moderate to highest levels of stress. Significant differences in the comparison of students with moderate to highest distress and students with no or little distress were identified linked to the field of studies. 50.0% (n = 137) of students enrolled in business-related studies reported moderate to highest levels of distress and 39.7% (n = 252) reported no or little stress. In health- and MINT/Technical-related subjects, the majority of students reported no or little stress level (17.5% vs. 13.5% and 19.2% vs. 9.9%; p < 0.001; chi square test). Details can be found in Table 3.

**Table 3:** Stress level due to financial difficulties.

Field of studies	Moderate to highest stress level (n = 274) n (%)	No or little stress level (n = 636) n (%)	P-value
business	137 (50.0%)	252 (39.7%)	<0.001
health	37 (13.5%)	111 (17.5%)	
IT	43 (15.7%)	106 (16.7%)	
MINT/technical	27 (9.9%)	122 (19.2%)	
other	30 (10.9%)	44 (6.9%)	
missing	0	1	

IT: Information technologies; MINT: mathematics, informatics, natural sciences and technology

A comparison of students with moderate to highest distress and students with no or little distress showed that those who worked part-time before the onset of the pandemic reported higher stress (65.7%; n = 178) compared to those who did not work part-time before the onset of the pandemic (34.3%; n = 93; p < 0.001; chi square test). Significant differences were identified in students who reported their financial limitations had increased with the pandemic. 71.5% (n = 196) of those with higher distress reported increased financial limitations compared to those with no or little stress (24.3%; n = 154; p < 0.001; chi square test).

### Association between students’ distress and secondary economic outcomes

In terms of participants’ change in income, significant differences were identified. Students reporting that their income had decreased, had significantly higher distress compared to those whose income did not decrease. This was especially notable among students whose income had decreased very much (29.9%; n = 82 vs. 1.1%; n = 7) or much (16.4%; n = 45 vs. 4.4%; n = 28; p < 0.001; Wilcoxon test). Further significant differences were identified in comparing students’ stress levels and changes in spending. Students with moderate to high levels of negative stress decreased their weekly spending (very) much compared to students with no or little negative stress.

Planned decrease in spending in the near future (weeks or months) was reported significantly more often by students with higher stress (very) much (35.9%; n = 98 vs. 6.7%; n = 42; p < 0.001; Wilcoxon test). Also, students with moderate to high distress reported significantly more (strong) negative consequences of the pandemic on their internship, job search and employment opportunities (74.0%; n = 202 vs. 46.3%; n = 294; p < 0.001; Wilcoxon test).

### Risk factors for students’ increase in financial limitations

In terms of students’ increase in financial limitations, no significant difference was found between male and female students (p = 0.509; chi square test). However, relevant differences relating to students’ field of studies were identified. Students who reported that financial limitations had increased were significantly more often enrolled in business-related subjects compared to other subjects (50.0%; n = 177; p < 0.001; chi square test). Details can be found in Table 4.

**Table 4:** Comparison of financial limitations and field of studies.

Are you postponing purchases to the future?	No (n = 562) n (%)	Yes (n = 354) n (%)	P-value
Business	214 (38.1%)	177 (50.0%)	<0.001
Health	87 (15.5%)	61 (17.2%)	
IT	104 (18.5%)	45 (12.7%)	
MINT/technical	124 (22.1%)	29 (8.2%)	
Other	32 (5.7%)	42 (11.9%)	
Missing	1	0	

IT: Information technologies; MINT: mathematics, informatics, natural sciences and technology

Students who worked part-time before the beginning of the pandemic reported significantly more often an increase in financial limitations due to the pandemic compared to those who did not work part-time (67.0%; n = 233 vs. 33.0%; n = 115; p < 0.001; chi square test). Students who reported an increase in financial limitations due to COVID-19 disclosed significantly more often moderate to highest stress (56.0%; n = 196) compared to those with no or little stress (44.0%; n = 154; p < 0.001; chi square test). Students with increased financial limitations reported significantly more often (strong) negative consequences of the pandemic on the job market (71.2%; n = 252) compared to those without increased financial limitations (44.3%; n = 247; p < 0.001; Wilcoxon test).

### Economic outcome differences between male and female students

An analysis of results stratified by gender related to a potential decrease in income, identified a significant difference between males and females. More female students reported a decrease in income compared to male students (40.4%; n = 190 vs 31.0%; n = 138; p = 0.032; Wilcoxon test). Significant differences were found in relation to a comparison of part-time work: Female students worked more in the categories of 1 to 12 hours compared to male students (29.4%; n = 136 versus 18.9%; n = 79). A comparison of those who worked most hours (more than 20 hours) per week, more male students worked part-time before the beginning of the pandemic: 22.1% (n = 93) male students versus 15.8% (n = 73) female students (p = 0.022; Wilcoxon test). No significant differences were identified related to gender and level of negative stress due to financial difficulties (p = 0.366; t test). In terms of a potential decrease in future spending, females decreased their spending significantly more compared to males: 60.5%; n = 280 versus 48.5%; n = 207; p = 0.005; Wilcoxon test). Anticipated consequences (negative or positive) on students’ internship, job search and employment opportunities, showed that significantly more female students assumed negative consequences: 58.5% (n = 275) compared to 50.6% (n = 218) males (p = 0.043, chi-squared test).

### Multivariate regression of increase in stress level and financial limitations

Multivariable logistic regression assessed risk factors for financial distress are shown in Table 5. The odds ratio for work part-time was 1.811 with p = 0.003 revealing that participants working part-time had a higher probability of financial distress compared to participants not working part-time. Also, the odds ratio for regular income before the pandemic was 0.456 with p = 0.014, indicating that students with regular income were less likely to experience financial distress compared to participants with no income (“I live from savings”). Differences with regard to university location and field of study were identified. The AUC of the logistic regression model amounted to 0.699 (95%-CI = [0.655, 0.742]). The assessment of variance inflation factors to check for multicollinearity revealed no multicollinearity issue, with all VIFs being below 1.74.

**Table 5:** Logistic regression results for financial distress.

	Odds ratio	95% Confidence interval	P-value
Gender: male vs. female (ref.)	0.974	[0.672, 1.412]	0.888
Age (years)	1.037	[0.987, 1.09]	0.148
Site: Ludwigshafen vs. Heidelberg (ref.)	0.598	[0.374, 0.955]	0.031
Site: Mannheim vs. Heidelberg (ref.)	0.313	[0.191, 0.514]	<0.001
Studies: health vs. business (ref.)	0.291	[0.161, 0.523]	<0.001
Studies: IT vs. business (ref.)	0.922	[0.558, 1.522]	0.75
Studies: MINT/technical vs. business (ref.)	0.667	[0.369, 1.205]	0.18
Studies: other vs. business (ref.)	1.325	[0.713, 2.462]	0.373
Part-time working vs. no part-time working (ref.)	1.811	[1.225, 2.676]	0.003
Grade: good vs. very good (ref.)	1.037	[0.67, 1.605]	0.871
Grade: average vs. very good (ref.)	1.261	[0.736, 2.159]	0.399
Grade: poor vs. very good (ref.)	2.426	[0.413, 14.237]	0.326
Semester	0.961	[0.875, 1.055]	0.399
Income before (irregular income) vs. no income (ref.)	0.486	[0.184, 1.282]	0.145
Income before (regular income) vs. no income (ref.)	0.456	[0.245, 0.851]	0.014
Degree: master vs. bachelor (ref.)	0.977	[0.582, 1.64]	0.93

IT: Information technologies; MINT: mathematics, informatics, natural sciences and technology

Multivariable logistic regression assessed risk factors for financial limitations are shown in Table 6. The odds ratio for working part-time was 2.094 with p<0.001, meaning that participants who worked part-time have a higher probability of increased financial limitations compared to participants without part-time working. Differences with regard to university location and field of study were identified. The AUC of the logistic regression model amounted to 0.755 (95%-CI = [0.718, 0.792]). The assessment of variance inflation factors to check for multicollinearity revealed no multicollinearity issue, with all VIFs being below 1.75.

**Table 6:** Logistic regression: Financial limitations analysis.

	Odds ratio	95% Confidence interval	P-value
Gender: male vs. female (ref.)	1.281	[0.882, 1.861]	0.194
Age (years)	1.037	[0.987, 1.089]	0.146
Site: Ludwigshafen vs. Heidelberg (ref.)	0.459	[0.288, 0.733]	0.001
Site: Mannheim vs. Heidelberg (ref.)	0.183	[0.111, 0.302]	<0.001
Studies: health vs. business (ref.)	0.42	[0.247, 0.714]	0.001
Studies: IT vs. business (ref.)	0.517	[0.309, 0.865]	0.012
Studies: MINT/technical vs. business (ref.)	0.39	[0.214, 0.71]	0.002
Studies: other vs. business (ref.)	1.362	[0.723, 2.567]	0.339
Part-time working vs. no part-time working (ref.)	2.094	[1.427, 3.075]	<0.001
Grade: good vs. very good (ref.)	1.405	[0.911, 2.168]	0.124
Grade: average vs. very good (ref.)	1.316	[0.765, 2.264]	0.321
Grade: poor vs. very good (ref.)	1.995	[0.316, 12.609]	0.463
Semester	0.932	[0.851, 1.022]	0.133
Income before (irregular income) vs. no income (ref.)	0.462	[0.172, 1.242]	0.126
Income before (regular income) vs. no income (ref.)	0.61	[0.323, 1.152]	0.127
Degree: master vs. bachelor (ref.)	0.965	[0.573, 1.624]	0.892

IT: Information technologies; MINT: mathematics, informatics, natural sciences and technology

## Discussion

This study examined effects of the COVID-19 pandemic on the economic situation of university students in Germany with a focus on financial limitations and financial distress and associated risk factors. The COVID-19 pandemic had both negative health-related and economic consequences on study participants. Findings demonstrated that more than one third of participants had decreased income. Financial constraints increased and economic uncertainty was experienced as a result of the pandemic.

Economic uncertainty during the COVID-19 pandemic has been shown to have a positive correlation with job uncertainty and a negative correlation with wellbeing [39]. In Japan, almost 50% of working students lost their employment due to the pandemic, affecting their lives, studies and health, resulting in the need to offer a safety net for underprivileged students [40]. Consequently, more than one third of students were concerned with living costs and university fees and those being aware of their financial insecurity reported poor health [40]. These findings correspond to students’ responses in our study with their increased financial limitations, reduced weekly consumption and the planned reduction of future expenditures. Findings from US American students showed that caused by the current pandemic, 13% of students postponed graduation, 40% lost employment, internship or offer and 29% expected to earn less at the age of 35 (15 years after the onset of the pandemic) [28]. Also, students who had lower incomes were 55% more likely compared to higher-income students to have delayed graduation due to COVID-19 [28].

In our study, decreased income and increased financial limitations were associated with higher distress. Those students with sources of regular income experienced less financial distress but results showed that students with decreased income, particularly those with only part-time work, were at risk of increased financial distress and pressure to modify consumptions patterns. Half of this group of students were from business programs. Work-related stressors such as high job insecurity were linked with poor mental health of young workers [41]. Further US American research reported that those who became unemployed after March 1, 2020 (compared to those who kept their job) stated more than twice as many days of being mentally ill [42]. Those in the lowest household income groups (prior to the pandemic) reported worst mental health [42]. A correlation between poor mental health and job insecurity and job loss is reciprocal: Mental ill health among students is linked to impaired academic success, lower levels of occupational preparedness and poorer future occupational performance [43]. In addition, work-related stressors such as high job insecurity have been linked to poor mental health among young workers [41]. Financial hardship and economic uncertainty went hand-in-hand with distress and subsequent negative impacts on mental health.

Gender-related differences concerning students’ financial situation were identified in this study. Gender differences related to job insecurity and mental health have been also reported in the literature: Women had a 24% higher chance to permanently lose their job compared to men, women expected a decrease in income by 50% more compared to men. Also, women were more likely to reduce consumption and raise savings [44]. Also, females and those without social support systems reported significantly poorer mental health outcomes [42]. Yet, women adopted more coping strategies that led to more positive mental health outcomes [45]. Financial challenges impacted more female students during the COVID-19 pandemic.

### German response to student financial hardship

To ease financial distress in pandemic or other emergency situations, a temporary loan might offer vulnerable students a certain amount of relief. In Germany, information on interest-free student loans, such as the KfW (*Kreditanstalt für Wiederaufbau*) student loan, could be shared widely to offer students ways to alleviate financial hardship and to secure continued basic needs [46]. A total of 100 mEUR in interim aid was available from the federal government for the months of June, July and August 2020 [47]. Yet, criticism has been raised as interest was only waved for several months and the loan was not accessible to anyone 45 years or older, in the 11th semester or higher or who was studying at vocational/apprenticeship academies [48]. In addition, students recipients are required to start paying off these loans by October 2022, which can only be postponed if the credit is used for a longer period [48]. The interest free period was to attract students but there would be difficulty with repayment for those who lost their part-time job during the pandemic to pay back a loan starting from October 2022 [47]. The Free Democratic Party university expert, Jens Brandenburg, argued that the “KfW offered a half-hearted discount for new customers, but not a sustainable student loan” [49]. Further criticism addressed the maximum pay-out sum of 650 Euro which was considered “insufficient” in some cities [47]. From April 2021 the regular interest rate with an effective rate of 4.36% will apply, which is the highest rate of all providers of study credits [47]. Consumer advocates even called for urged caution as the repayment of debts begins in the middle of the rush hour of life when every cent is needed to start a family and live [47]. Despite the federal response to address financial hardship issues for students, this aid poses future financial challenges especially due to interest rates.

### Strengths and limitations

A key strength of this study was the response rate and the large amount of data from the Rhine-Neckar region, including universities and universities of applied sciences with 1,246 students, of which a total of 917 students successfully submitted the questionnaire. Nevertheless, this study has limitations that need to be considered when interpreting results. Firstly, the questions were not based on a validated questionnaire, but on a self-designed questionnaire built following a literature review. This represents a limitation with regard to direct regional or international comparisons. The second limitation is that convenience sampling was used. Convenience sampling does not provide representative results; therefore, the results of this study cannot be generalized to a wider population. Nonetheless, due to the high response rate, the results add useful knowledge and improve understanding of the key implications and challenges for students in the area who can help others design similar studies in other locations. The third limitation is due to the cross-sectional study design, thus not allowing to assess distress and increased financial limitations over the course of time, thus not allowing to model causal effects of the pandemic. A further limitation may consider the fact that the survey was conducted in English language only and students’ command of English language skills was not verified prior to the survey participation.

### Conclusion

Negative consequences of the COVID-19 pandemic on the economic situation of students were identified in this cross-sectional survey. The most prominent risk factors for financial distress and increased financial limitations were working part-time and no regular income. University students were disproportionally affected by the economic consequences of the COVID-19 pandemic, which increased students’ economic uncertainty. To offset such problems, financial aid schemes for students need to be made available to alleviate distress and to allow students to focus on their studies but should not compound problems by leading to financial hardship at a later point in time. Also, implications for university education and psychology practice (e.g. proactive risk-stratification, early screening programs, financial counseling and mental health support programs) should be assessed to alleviate students’ distress.

## Supporting information

S1 Checklist (PDF)

S1 File (DOCX)

## Abbreviations

AUC: Area under the curve
BAföG: Bundesausbildungsförderungsgesetz (Federal Education and Training Assistance Act)
CI: Confidence interval
COVID-19: Coronavirus disease 2019
IQR: Interquartile range
IT: Information technology
KfW: Kreditanstalt für Wiederaufbau (KfW Banking Group)
MINT: Mathematics, Informatics, Natural Sciences and Technology
OR: Odds ratio
SD: Standard deviation
VIF: Variance inflation factor
