# Supplementary material for: “University students’ economic situation during the COVID-19 pandemic: A cross-sectional study in Germany”
Source: PLoS One. 2022 Oct 6;17(10):e0275055. doi: 10.1371/journal.pone.0275055 (PMC9536534; doi:10.1371/journal.pone.0275055)
Supplement: S1 File — (DOCX) [file pone.0275055.s002.docx]

**Supplementary material**

Table 1: Income prior to the pandemic

| **Have you had any income before the COVID-19 pandemic? n (%)** | |
| --- | --- |
| no income, I live from savings | 70 (8.4%) |
| yes, irregular income | 36 (4.3%) |
| yes, regular income from family | 100 (12.0%) |
| yes, regular income from family and irregular income | 19 (2.3%) |
| yes, regular income from a loan | 70 (8.4%) |
| yes, regular income from BAföG | 23 (2.8%) |
| yes, regular income from (student) job | 324 (38.8%) |
| yes, regular income from (student) job and family | 108 (12.9%) |
| yes, regular income from (student) job and BAföG | 18 (2.2%) |
| other combination | 66 (7.9%) |
| missing | 83 |

BAföG: Bundesausbildungsförderungsgesetz i.e. Federal Education and Trainings Assistance Act

Table 2: Employment prior to the pandemic

| **Before the beginning of the COVID-19 pandemic in Germany, did you work part-time? n (%)** | |
| --- | --- |
| yes, 1-4 hours per week | 60 (6.7%) |
| yes, 5-8 hours per week | 88 (9.9%) |
| yes, 9-12 hours per week | 68 (7.6%) |
| yes, 13-16 hours per week | 38 (4.3%) |
| yes, 17-20 hours per week | 84 (9.4%) |
| yes, more than 20 hours per week | 168 (18.8%) |
| I tried to get a part-time job but was not accepted | 43 (4.8%) |
| no, I did not want to work as I want to focus on my studies | 344 (38.5%) |
| missing | 24 |

Table 3: Postponement of purchases

| **Are you postponing purchases to the future? n (%)** | |
| --- | --- |
| no, I am not postponing any purchases | 454 (49.7%) |
| yes, any purchases which are not strictly necessary (such as food/grocery shopping) | 199 (21.8%) |
| yes, purchases above 20 Euro | 26 (2.8%) |
| yes, purchases above 50 Euro | 55 (6.0%) |
| yes, purchases above 100 Euro | 62 (6.8%) |
| yes, purchases above 200 Euro | 63 (6.9%) |
| yes, purchases above 500 Euro | 29 (3.2%) |
| yes, purchases above 1000 Euro | 25 (2.7%) |
| missing | 4 |

Table 4: Change in future spending

| **Are you planning to decrease your spending in the next weeks or months? n (%)** | |
| --- | --- |
| very much | 53 (5.9%) |
| much | 87 (9.6%) |
| a little | 297 (32.9%) |
| very little | 58 (6.4%) |
| not at all | 407 (45.1%) |
| missing | 15 |
